# Supplementary material for: Healthcare providers’ palliative care graded diagnosis and treatment behavior, attitudes, self-efficacy, compassion fatigue, and workplace well-being: A mediating moderation model
Source: PLoS One. 2025 Nov 21;20(11):e0336925. doi: 10.1371/journal.pone.0336925 (PMC12637986; doi:10.1371/journal.pone.0336925)
Supplement: Attachment — Submitted filename: Response to Reviewers.doc [file pone.0336925.s002.doc]

**Reviewer #1: thank you for your effort to modify your article in form that improvment make it very useful in increasing body of knowlege thanks again and keep going , but i finnaly recommend minir english revision to improve rediability of your manuscript**

**Response:** Thank you very much for your kind words and valuable feedback. I have completed the minor English revisions as you suggested to further enhance the readability of the paper. Your feedback has been incredibly helpful in refining the manuscript, and I am grateful for your support. Thank you once again for your guidance!

**Reviewer #2: Thanks for inviting me for reviewing this topic Please consider the following:**

1. **clarify more practical implications for nursing in abstract and implications section**

**Response:** We would like to express our sincere gratitude to the reviewer for the valuable feedback, particularly the suggestion to further elaborate on the practical significance for nursing in both the abstract and implications sections. Your comments have greatly assisted us in clearly articulating the practical applications of this study, especially in how enhancing healthcare professionals' self-efficacy can optimize the grading of palliative care and improve nursing quality. Your insights have significantly contributed to refining and deepening the discussion of the study's significance, playing a crucial role in the improvement of the research. We sincerely appreciate your thoughtful input.

**2. introduction: the significance of the study need to be highlighted and more clarified**

**Response:** Thank you for your valuable feedback. I appreciate your suggestion regarding the need to further highlight and clarify the significance of the study. In response, I have added additional clarification in the final paragraph of the introduction.

**The revised content is as follows:**

“In conclusion, self-efficacy plays a significant role in healthcare professionals' attitudes and behaviors towards graded treatment, compassion fatigue, and well-being at work. While the role of self-efficacy in nursing has been acknowledged, its mechanisms within the context of graded treatment remain unclear. Ignoring self-efficacy could lead to a lack of motivation among healthcare professionals in clinical decision-making and work execution, thus affecting the quality and efficiency of healthcare services. Additionally, neglecting the potential impact of self-efficacy on healthcare professionals' psychological states and behaviors may exacerbate burnout, reduce job satisfaction, and weaken professional performance, ultimately impacting patient experience and the optimization of the healthcare system.

Therefore, this study aims to investigate whether self-efficacy mediates the relationship between healthcare professionals' attitudes and behaviors towards graded treatment and to evaluate a moderated mediation model. We hypothesize that self-efficacy may mediate the relationship between attitudes and behaviors, and also moderate the indirect effect of compassion fatigue on well-being at work.”

**3. clarify more the rationale for selecting such settings with detailed description.**

**Response:** Thank you for your valuable feedback. I appreciate your suggestion regarding the need to clarify the rationale for selecting the study settings. In response, I would like to explain that the hospital in Wuhan was chosen due to its advantageous geographical location, large scale, and extensive case pool, which made sample collection convenient. Additionally, as I work in this hospital, it provided a more accessible and practical setting for conducting the study.

On the other hand, the tertiary hospital in Huangshi, a non-provincial capital city, was included to ensure the study covered healthcare institutions in smaller cities, which offers a broader regional perspective. This approach enhances the study’s applicability to a wider range of healthcare settings. To collect data, I employed convenience sampling, which ensured the feasibility of gathering the sample while allowing for a comprehensive understanding of the healthcare services in different regions.

**4. reliability and pilot study clarify whether it is from the same study hospital or different one?**

**Response:** Thank you for your valuable feedback. Regarding your inquiry about reliability and the pilot study, I would like to clarify that the pilot study was conducted at both the Wuhan and Huangshi hospitals, which were the same hospitals used for the main data collection. If you believe it is necessary to explicitly highlight this in the article, I will promptly make the necessary adjustments and provide further clarification.

**5.Implications for practice need to be modified to more applicable actions**

**Response:** Thank you for your feedback. I have made revisions based on your suggestions. Below is the updated section on implications for practice.

**The revised content is as follows:**

“In conclusion, this finding provides valuable insights for clinical practice. Enhancing healthcare professionals' self-efficacy is key to successfully implementing tiered diagnosis and treatment. Through targeted training and interventions, boosting their confidence and decision-making abilities can effectively encourage their active participation in complex situations and improve the quality of tiered care. Hospitals should offer psychological support to alleviate stress and prevent burnout, thereby enhancing self-efficacy. Only by improving self-efficacy can healthcare professionals execute tasks more decisively and effectively in tiered care and palliative care, driving the rational allocation of resources and the integration of palliative care principles. Moreover, hospitals can optimize resource allocation, improve healthcare quality, and ultimately achieve the dual goals of enhancing patient experience and increasing healthcare professionals' job satisfaction by promoting policies, sharing best practices, and offering economic incentives.”

**6.clarify more strengths and limitations of the study**

**Response:** Thank you for your suggestion. I have expanded the content to provide a more comprehensive explanation of the study's strengths and limitations. The revised section addresses considerations such as potential confounding variables and sample representativeness, offering a more balanced perspective.

Reviewer #3: One point should be considered for using more updated references 2023-2024 in the literature.

1. **The sampling should be discussed in detail with references.**

**Response:** Thank you for your valuable feedback. According to the reference literature,when all the parameters are small and both the mediator and outcome are normal, approximately 371 observations are required to achieve a power of 0.8 for detecting a total indirect effect at the significance level of 0.05.

2. **The discussion should link all variables of the study with literature and results.**

**Response:** Thank you for your valuable feedback. I have revised the discussion section to better link all the variables in the study with both the relevant literature and the results. In the updated version, I have provided a more detailed analysis of each variable, comparing the findings with existing research to highlight similarities and differences.I appreciate your insightful suggestion, and it has been incorporated into the revision. Thank you once again for your guidance!

3. **More variables should be included in the study to make it stronger.**

**Response:** Thank you very much for your thoughtful suggestion. Currently, the study includes five key variables, and this selection was influenced by previous guidance I received, where it was emphasized that variables should be chosen based on positive indicators reported in the literature, rather than aiming to include as many variables as possible. The focus was placed on selecting the most relevant and meaningful variables for the research. That said, I deeply appreciate your input, and I will definitely keep your advice in mind as the study progresses. In the future, I hope to expand the scope of the study and consider adding more variables as the research develops. Thank you again for your valuable guidance!

4. **some paragraph need to add references.**

**Response:** Thank you for your valuable feedback. I have added references where necessary.

5. **You need to justify to make it clear to generalize your results.**

**Response:** Thank you again for your insightful suggestion. I have made the necessary revisions to the manuscript, where I have provided a clear justification for the generalizability of the study results. I have explained the scope and limitations of the study, along with the steps taken to ensure the validity and reliability of the findings. This should help clarify how the results can be applied to similar populations or settings. I appreciate your feedback, and I believe these changes strengthen the manuscript. Thank you once again for your valuable guidance!

6. **Data Collection need more details.**

**Response:** Thank you for your suggestion. Data were collected using an online survey platform generated from a dedicated URL link ( <https://www.wjx.cn/).> Survey WeChat QR code (a Chinese instant messaging app ) images were disseminated to the nursing program directors of the nine institutions, who also served as co-investigators.
