# Supplementary material for: Healthcare providers’ palliative care graded diagnosis and treatment behavior, attitudes, self-efficacy, compassion fatigue, and workplace well-being: A mediating moderation model
Source: PLoS One. 2025 Nov 21;20(11):e0336925. doi: 10.1371/journal.pone.0336925 (PMC12637986; doi:10.1371/journal.pone.0336925)
Supplement: Attachment — Submitted filename: Response_to_Reviewers_auresp_2.doc [file pone.0336925.s003.doc]

Reviewer #2: Areas for Improvement:
1. Abstract & Introduction
1.1 The abstract lacks clarity in describing key findings in a concise manner.
Recommendation: Summarize the major statistical results more succinctly while emphasizing the study’s novelty.
**Response:** Thank you very much for your kind words and valuable feedback. The content after our revisions is as follows: While prior research has acknowledged the role of self-efficacy in nursing, its specific mechanisms within graded treatment contexts remain underexplored. Our study investigates how self-efficacy bridges healthcare professionals' attitudes and behaviors towards graded treatment with compassion fatigue and workplace well-being.

Health care professionals' attitudes towards grading treatment predicted an increase in self-efficacy (β = 0.161, *p* < 0.001), which subsequently led to improved grading behavior in treatment (β = 0.647, *p* < 0.001). Self-efficacy mediated 34.81% of the effect of attitudes on grading behavior.
1.2 The introduction provides a broad background but could benefit from a stronger justification for why self-efficacy was chosen as a mediating/moderating factor.
Recommendation: Strengthen the theoretical underpinning for why self-efficacy is expected to influence both attitudes and behavior in palliative care.
**Response:** Thank you for your valuable feedback. The Health Action Process Approach (HAPA) emphasizes self-efficacy's role across all behavior - change stages. In the **motivational phase**, self-efficacy affects behavioral intention, influencing an individual's readiness for specific health behaviors[19]. In the **planning phase**, it motivates individuals to formulate detailed action plans and attempt the planned actions. In the **action phase**, self-efficacy helps maintain actions despite challenges and coordinates resource use to sustain healthy behaviors. Overall, self-efficacy mediates attitudes, intentions, and actual behaviors throughout the health behavior change process.
2. Methodology
2.1 The study relies entirely on self-reported measures, which can introduce bias.
Recommendation: Acknowledge this limitation more explicitly and suggest potential complementary methods, such as observational studies or supervisor evaluations.
**Response:** Thanks to the editor's suggestion, we've made the following changes:

**Limitations：**We acknowledge that the results obtained from the self-reported measures should be interpreted with caution due to the potential limitations inherent in such data collection methods.

**Conclusion：**Future studies should integrate observational methods (e.g., longitudinal interviews, retrospective cohorts) to complement self-report limitations and account for individual variability, enhancing research validity.

2.2 The justification for using SPSS PROCESS for mediation analysis is not clearly articulated. Recommendation: Briefly explain why this statistical approach was chosen over alternatives like Structural Equation Modeling (SEM).
**Response:** Thank you for your valuable feedback. We highly value your comments and have carefully reviewed the points raised. We’d like to clarify our choice of using the SPSS PROCESS macro for mediation analysis. Given the relatively simple mediation model in this study—focusing on a single mediator between the independent and dependent variables—SPSS PROCESS was deemed appropriate. It efficiently tests mediation effects and offers clear statistical results. Moreover, its seamless integration with SPSS makes it user-friendly, streamlining the analysis and interpretation process. Additionally, it provides various methods for mediation analysis, such as Sobel and Bootstrap, enhancing analytical flexibility. However, we acknowledge the usefulness of Structural Equation Modeling (SEM) and will consider it for future studies with larger sample sizes and more complex models.
3. Results & Interpretation
3.1 The mediation effect (34.81%) is moderate but not overwhelmingly strong. This nuance is not discussed.
Recommendation: Interpret the mediation effect in the context of previous studies—how does this compare with similar research?
**Response:** Thank you so much for your insightful feedback. In a study conducted at 10 compulsory isolation drug rehabilitation centers in Zhejiang Province, 1,197 Substance use disorders (SUDs) patients were recruited. The findings indicated a positive effect of attitudinal behavior on behavioral intention (β = 0.02), while self-efficacy did not emerge as a mediator. In contrast, self-efficacy appeared as a mediating variable in this study, with the nurse population and showed a large effect size (34.81%). Nurses, as professionals, may derive their self-efficacy from their education, training, and work experience, which enhances their confidence in their abilities. Higher self-efficacy is essential for nurses to overcome the challenges and pressures of their work, making it a crucial factor in forming their behavioral intentions. Different behavioral intentions may require varying levels of self-efficacy to support them; for instance, nurses' behavioral intentions may relate to the provision of care and interventions, whereas SUD patients'.
3.2 While the moderation model is statistically significant, the practical significance (effect size) is not explicitly discussed.
Recommendation: Provide effect size measures to contextualize the practical importance of the findings.
**Response:** Thank you for your valuable feedback. I have now explicitly discussed the practical significance of the findings by providing effect size measures. In the revised section, I have included a detailed analysis of the effect size, which shows that the mediation effect of self-efficacy on the relationship between healthcare providers' attitudes and their tiered diagnosis and treatment behaviors accounts for 34.81% of the total effect. This effect size underscores the practical importance of self-efficacy in shaping healthcare providers' attitudes and behaviors, thereby supporting Hypothesis 3. I believe this addition provides a clearer understanding of the practical implications of the study results.
4. Discussion & Implications
4.1 The discussion restates results but lacks deeper engagement with alternative explanations.
Recommendation: Consider potential confounders, such as institutional culture or workload, that may have influenced results.
**Response:** Thank you for your valuable feedback. We have carefully considered your suggestion regarding the need for deeper engagement with alternative explanations in the discussion section. In response, we have revised the discussion to include a more thorough examination of potential confounders that may have influenced our results. Specifically, we have now incorporated an analysis of institutional culture and workload as possible factors that could have affected the outcomes observed in our study. We believe that this enhanced discussion provides a more comprehensive understanding of our findings and acknowledges the complexity of the factors at play.

4.2 The discussion does not address cultural factors in China that may impact attitudes toward palliative care. Recommendation: Include a brief discussion on cultural attitudes towards end-of-life care and whether findings might differ in other healthcare systems.
**Response:** Thank you so much for your insightful feedback. In mainland China, death has historically been regarded as a taboo, influenced by Confucian, Taoist, and Buddhist philosophies as well as superstitious beliefs[40]. This cultural context has engendered a pervasive avoidance of death-related topics, which in turn has fostered negative attitudes among physicians toward triage behavior in healthcare professionals.

5. Limitations & Future Directions
The limitations section is well-structured but could mention response bias more explicitly. Recommendation: Suggest future studies incorporating mixed-methods approaches to mitigate self-reporting bias.

**Response:** Thank you for your valuable feedback. We acknowledge that the results obtained from the self-reported measures should be interpreted with caution due to the potential limitations inherent in such data collection methods. Future studies should integrate observational methods (e.g., longitudinal interviews, retrospective cohorts) to complement self-report limitations and account for individual variability, enhancing research validity.

Reviewer #4: 
1. The title is very critical and important, additionally it covers an extra point of research not covered in previous research.

**Response:** Thank you for your positive feedback regarding the title. I have carefully reviewed the content and can confirm that all the relevant aspects are indeed covered. If there are any other concerns or suggestions, please do let us know. We are open to feedback and will take your comments seriously.
2. Methodology: Please mention more specifically why select these two hospitals

**Response:** We deeply appreciate the valuable feedback you've given. The hospitals in Wuhan represent the palliative care triage levels in provincial cities, while those in Huangshi reflect the triage levels in regional cities. The number of beds in these two hospitals ranges from 1,800 to 3,300.
3. Data collection process very short give more clarification about actual process regarding the duration to fill every sheet, additionally how you decrease the bias during data collection process, Moreover, more clarification about the role of co-investigators

**Response:** Your feedback is greatly valued and has been instrumental to our work. **Data collection methods** The researcher contacted the hospital, secured approval from its nursing department, and held face-to-face meetings with the potential participants and hospital director to explain the study’s purpose, risks, and benefits. Trained research assistants from each hospital collected data following a standardised protocol and served as co-investigators. Data were gathered via an online survey platform (<https://www.wjx.cn/>) . To ensure data integrity, each questionnaire could be submitted only once per IP address. Participants were assured of confidentiality, anonymity, and their right to withdraw at any time without consequences.

4.The Palliative Care Graded Diagnosis and Treatment Knowledge, Attitudes, and Practices tool please tension about this paragraph “In the knowledge dimension, participants were primarily surveyed on their knowledge of palliative care graded diagnosis and treatment, rated on a 5-point scale (1 = strongly disagree, 5 = strongly agree). Higher scores indicate more positive attitudes towards palliative care graded diagnosis and treatment. The behavior dimension consists of 13 items, rated from 1(very rarely) to 5(always), with higher scores indicating more proactive engagement in palliative care graded diagnosis and treatment. “ please more clarification about the three dimensions in this tool about how many items in each dimension because you mention knowledge dimension and you mention this dimension used to assess attitude and you didn’t mention the attitude dimension itself and how many items????.

**Response:** Thank you for your valuable feedback. In the knowledge dimension, contains 11 entries with single-choice answers, participants were primarily surveyed on their knowledge of palliative care triage. The attitude dimension had 22 items, rated on a 5-point scale (1 = strongly disagree, 5 = strongly agree). Its total score ranged from 22 to 110, with higher scores indicate more positive attitudes towards palliative care triage. The behavior dimension consists of 13 items, rated from 1( very rarely) to 5(always),had a total score range of 13 to 65. Higher scores indicating more proactive engagement in palliative care triage.

5、In the General Self-Efficacy Scale (GSES), developed by Ralf Schwarzer, was used to measure nurses’ self-efficacy , you didn’t mention the Ralf Schwarzer article in the reference section
**Response:** Your constructive feedback is highly appreciated. 34. Zhang JX, Schwarzer R. Measuring optimistic self-beliefs: a Chinese adaptation of the general self-efficacy scale. Psychologia: An International Journal of Psychology in the Orient. 1995;:174–81.
6. What about pilot sample ?

**Response:** Prior to the formal implementation of this study, we conducted a pilot study to assess the feasibility of the questionnaire, the response of the participants, and the effectiveness of the data collection process. The pilot study was carried out in June 2023, involving 60 nurses from the same two hospitals that were included in the main study. The purpose of the pilot was to identify and address any issues that could potentially affect the outcomes of the formal study, including the difficulty of understanding the questionnaire, the level of participant engagement, and the integrity of the data collected.The results of the pilot study indicated that the questionnaire was generally clear and that participants were able to understand and complete all items. However, we observed that several items in the General Self-Efficacy Scale required further clarification to ensure that all participants could accurately comprehend them. Additionally, the preliminary analysis of the pilot data suggested that the sample size might not be sufficient to detect subtle differences between self-efficacy, compassion fatigue, and job satisfaction. Consequently, through a calculation of the required sample size, we decided to increase the sample size for the formal study to 900 nurses to enhance the statistical power.

7.This manuscript requires additional editing and proofreading to correct detected errors
**Response:** Thank you for your constructive feedback regarding the manuscript. We have taken your comments seriously and have conducted additional editing and proofreading to address the errors that were detected. We are confident that the manuscript has been significantly improved in terms of language accuracy and overall clarity.We would be grateful if you could review the revised manuscript and provide us with any further suggestions or comments you may have to ensure the highest quality of our submission.

8. Please add DOI in some references

**Response:** Thank you for your attention to detail. We have added the DOIs to the relevant references as suggested. Additionally, we have conducted a thorough check to ensure that all references now include the appropriate DOI information where available.
