# Supplementary material for: Healthcare providers’ palliative care graded diagnosis and treatment behavior, attitudes, self-efficacy, compassion fatigue, and workplace well-being: A mediating moderation model
Source: PLoS One. 2025 Nov 21;20(11):e0336925. doi: 10.1371/journal.pone.0336925 (PMC12637986; doi:10.1371/journal.pone.0336925)
Supplement: Attachment — Submitted filename: review.docx [file pone.0336925.s004.docx]

The study addresses a highly relevant and underexplored topic, offering valuable insights into the psychosocial dynamics affecting palliative care practices among nurses. The use of a moderated mediation model enhances the conceptual strength of the manuscript. However, several aspects would benefit from improved clarity, methodological detail, and consistency in reporting. The following specific comments are intended to support the refinement of your manuscript.

- The manuscript inconsistently uses the terms “grading treatment,” “graded diagnosis and treatment,” and “triage.” Therefore standardize terminology throughout. If "graded diagnosis and treatment" is a unique model in China, define it clearly early on and use it consistently (e.g., "graded treatment"). If you intend to use "triage" as a broader concept, clarify its equivalence or difference with "graded treatment."
- Condense the aims into a single, cohesive paragraph. For example: "This study aims to (1) examine the relationship between healthcare professionals’ attitudes and behaviors regarding palliative care grading treatment and the mediating role of self-efficacy, and (2) explore whether self-efficacy moderates the effect of compassion fatigue on well-being at work."
- Improve Background Flow by streamline by grouping related ideas (e.g., barriers to palliative care delivery, role of healthcare professionals, system-level challenges) into more cohesive thematic sections. You can use updated studies in the background “Toqan D, Malak MZ, Ayed A, Hamaideh SH, Al-Amer R. Perception of nurses’ knowledge about Palliative Care in West Bank/Palestine: levels and influencing factors. Journal of palliative care. 2023 Jul;38(3):336-44”.

“Awad B, Batran A, Malak MZ, Ayed A, Shehadeh A, Alassoud B, Ejheisheh MA. Knowledge, attitudes, and self-efficacy regarding palliative care among Palestinian nurses in intensive care units. BMC nursing. 2025 Apr 17;24(1):435”.

- Sharpen the Justification for the Study "To our knowledge, this is among the first studies to assess both the mediating and moderating roles of self-efficacy in the context of palliative care treatment grading in China, filling a critical gap in understanding the psychosocial mechanisms behind healthcare professionals' behaviors."
- Change “To investigates” to “To investigate” in Aims. Change “triage attitude” to “attitudes toward graded treatment” (if this is the consistent term).
- Design ???
- Population and procedure: The text mentions distributing 900 surveys and completing 890, but later also says 900 were distributed and 742 were valid—consider clarifying this sequence for better coherence.
- Ethical approval : Clarify confidentiality procedures: You may elaborate slightly on how identification numbers were used (e.g., coded, encrypted).
- Measurement tools: Cronbach’s alpha placement: Mention the reliability coefficient at the end of each scale section for clarity.
